# Genetic Deletion of NP1 Prevents Hypoxic‐Ischemic Neuronal Death Via Reducing AMPA Receptor Synaptic Localization in Hippocampal Neurons

**DOI:** 10.1161/JAHA.112.006098

**Published:** 2013-02-22

**Authors:** Md Al Rahim, Mir Ahamed Hossain

**Affiliations:** 1 Hugo W. Moser Research Institute at Kennedy Krieger, Baltimore, MD (M.A.R., M.A.H.); 2 Department of Neurology, Johns Hopkins University School of Medicine, Baltimore, MD (M.A.R., M.A.H.)

**Keywords:** AMPA receptors, neuronal pentraxin 1, oxygen glucose deprivation, receptor trafficking, surface expression, synaptic clustering

## Abstract

**Background:**

Trafficking of α‐amino‐3‐hydroxy‐5‐methyl‐4‐isoxazole propionic acid receptors (AMPARs) to excitatory synapses is critical to their synaptic functions. Previously, we have shown induction of neuronal pentraxin 1 (NP1) and its colocalization with AMPAR subunit GluR1 in hypoxic‐ischemic (HI) brain injury. However, the role of NP1 in mediating GluR1 surface expression, trafficking, and clustering at synapses in HI neuronal death is unclear.

**Methods and Results:**

Primary hippocampal neurons, isolated from wild‐type (WT) and NP1‐knockout (C57BL/6 background) mice at DIV 12 to 14 were exposed to 2 to 8 hours of oxygen glucose deprivation (OGD)—in vitro conditions that mimic human stroke. OGD exposure resulted in time‐dependent induction of NP1 (∼4‐fold), enhanced redistribution of AMAP GluR1 receptors at excitatory synapses, and increased neuronal death. We observed a significant increase in surface and synaptic GluR1 clusters that colocalized with PSD‐95 on dendrites with a simultaneous decrease in internalized GluR1. Surface cross‐linking with BS^3^ showed enhanced membrane insertions of GluR1, and increased phosphorylation at Ser‐845 further supported enhanced surface availability of GluR1 after OGD. NP1 protein colocalized with GluR1 and PSD‐95, and OGD significantly increased their synaptic coclustering. Most strikingly, the genetic deletion of NP1 resulted in decreases in surface GluR1 cluster density, synaptic localization, phospho‐GluR1 (Ser‐845) levels, and neuronal death after OGD compared with WT neurons. AMPA (50 μmol/L) induced NP1 and significant cell death in WT but not in NP1−/− neurons.

**Conclusions:**

Our results indicate that NP1 plays a key role in synaptic clustering of GluR1, suggesting that targeting NP1 might be a practical approach to preventing ischemic brain damage.

## Introduction

Glutamate is the major excitatory neurotransmitter in the central nervous system (CNS) and plays a key role in maintaining normal physiological processes, including neural development, excitatory synaptic transmission, and plasticity.^1^ The α‐amino‐3‐hydroxy‐5‐methyl‐4‐isoxazole propionic acid receptor (AMPAR) and *N*‐methyl‐d‐aspartic acid (NMDA) receptor are the main excitatory glutamate receptors in the mammalian brain and cluster on postsynaptic neural membranes.^2^ Glutamate accumulates at synapses immediately following hypoxia‐ischemia (HI), resulting in excessive stimulation of glutamate receptors,^3^ which is considered a primary intracellular event that induces neuronal cell death in the brain.^4–6^ Furthermore, the neonatal brain is far more susceptible to excitotoxicity than the adult brain.^4–5^ Therefore, regulation of glutamate receptor function and/or synaptic localization could be a practical approach to prevent HI neuronal injury.

The clustering of postsynaptic glutamate receptors is considered one of the earliest events in excitatory synapse formation,^2,7^ and AMPAR trafficking at postsynaptic membranes is critical for synaptic strength and efficacy.^8^ At excitatory synapses of central neurons, inotropic glutamate receptors including AMPARs are organized into multiprotein signaling complexes within the postsynaptic density (PSD).^9^ One of the synaptic PDZ domain proteins, PSD‐95, has been intensively studied for its possible role in the clustering of receptors and channels.^10^ PSD‐95 binds many key constituent proteins such as NMDAR^11^ and AMPAR^12^ and plays a dominant role in controlling AMPAR numbers at synapses.^13^ However, how PSD‐95 in combination with other proteins actually controls AMPAR synaptic localization during ischemia is still not well understood. On the other hand, candidates for AMPAR‐clustering factor include members of the neuronal pentraxin family; neuronal pentraxin 1(NP1), neuronal activity‐regulated pentraxin (Narp; also called NP2), and neuronal pentraxin receptor.^14–15^ The neuronal pentraxins have several structural and functional characteristics that might play a role in promoting excitatory synapse formation and synaptic remodeling.^14–15^ Previously, we showed that NP1 colocalized with GluR1 and that hypoxia induced a time‐dependent increase in NP1–GluR1 interaction.^16^ However, the role of NP1 in regulating the membrane trafficking of GluR1 and its clustering at excitatory synapses in ischemic neuronal death remains to be investigated. Although glutamate receptors mediate ischemic brain damage^4,17–18^ and PSD‐95 is one of the most stable proteins in PSDs at excitatory synapses,^19^ blocking either of them could be deleterious as it may impair normal physiological functions of uninjured neurons.^20^ Therefore, it would be a practical approach of targeting interactive protein molecules that are involved in the trafficking of and forming clusters with glutamate receptors to suppress ischemic neuronal death.

In the present study, we have investigated the role of NP1 in synaptic GluR1 clustering in the progression of neuronal cell death following oxygen glucose deprivation (OGD). We show that NP1 colocalizes with GluR1 to form clusters on the cell surface and at synaptic sites and that OGD causes increased NP1 expression, GluR1 membrane insertion, and NP1‐GluR1‐PSD‐95 coclustering. In contrast, knocking down of the *NP1* gene prevents OGD‐induced enhanced synaptic GluR1 localization and neuronal cell death. Our findings identify NP1 as an important regulator of GluR1 membrane trafficking and synaptic clustering in the event of ischemic brain damage.

## Methods

### Hippocampal Neuronal Cultures

The Johns Hopkins University Institutional Animal Care and Use Committee approved all animal protocols used; it complied with the US NIH Guide for the Care and Use of Laboratory Animals. Primary hippocampal neuronal cultures were prepared from neonatal wild‐type (WT) and NP1‐knockout (NP1‐KO) mice (C57BL/6 background) on postnatal day 1 or 2, as described previously.^21^ NP1‐KO mice were kindly provided by Dr Paul Worley, Department of Neuroscience, School of Medicine, Johns Hopkins University. After plating, at 2 days in vitro (DIV), half the media were replaced with fresh medium also containing cytosine arabinofuranoside to a final concentration of 5 μmol/L to prevent nonneuronal proliferation. Thereafter, culture media was changed by half every 3 to 4 days. Cultures were used for experiments at 12 to 14 DIV. With this protocol, >95% of cultured cells were microtubule‐associated protein‐2 (MAP2)–immunoreactive neurons (Chemicon).^16^

### Induction of OGD

To induce OGD, primary hippocampal neurons cultured at DIV 12 to 14 were placed in glucose‐free Earl's balanced salt solution (EBSS) and exposed to humidified 95% N_2_/5% CO_2_ using anaerobic modular incubator chambers (Billups‐Rothenberg) for various periods (2 to 8 hours) at 37°C. Samples were processed immediately after OGD without any reoxygenation. Control cultures were placed in EBSS containing glucose and exposed to humidified 95% air/5% CO_2_ at 37°C for the same duration.^21–22^

### Assessment of Cytotoxicity

Immediately after the indicated periods of exposure, cytotoxicity was determined by lactate dehydrogenase (LDH) assay. LDH released into the media after OGD exposure was measured using a Cytotoxicity Detection Kit (Roche Diagnostics Corporation) as described previously.^16,21^ Percent cell death was determined using the formula: % cytotoxicity=OGD LDH release (OD_490_)/maximum LDH release (OD_490_) after correcting for baseline absorbance of LDH release at 490 nm.

### Assessment of Cell Viability

In the 3‐(4,5‐dimethylthiazol‐2‐yl)‐2,5‐diphenyl tetrazolium bromide (MTT) assay mitochondrial dehydrogenase activity cleaves MTT (Sigma) and is a biochemical index for cellular viability. A quantitative colorimetric assay of MTT was used to determine cell survival as described previously.^16,22^ The results were expressed as a percentage of control culture viability.

### Immunofluorescence

Surface AMPA receptors were measured as described by O'Brien et al^2,7^ and Wei et al.^23^ In brief, hippocampal cultures were fixed in 4% paraformaldehyde (20 minutes, room temperature) but not permeabilized. Neurons were incubated with a polyclonal anti‐GluR1 antibody (1:500; Millipore) overnight at 4°C. After washing, neurons were permeabilized and incubated with a monoclonal anti‐MAP2 antibody (1:250; Santa Cruz Biotechnology) for 2 hours at room temperature. Surface GluR1 was detected with Alexa Fluor 594 (red)–conjugated anti‐rabbit secondary antibody, whereas MAP2 was detected with Alexa Fluor 488 (green)–conjugated anti‐mouse secondary antibody. After washing in PBS 3 times, coverslips were mounted on slides with ProLong Gold Antifade with DAPI (Invitrogen). For the detection of AMPA receptors at synapses, neurons were fixed, permeabilized, and stained with a polyclonal anti‐GluR1 antibody (1:500; Millipore) and a monoclonal anti‐PSD95 antibody (1:500; Abcam) overnight at 4°C.

The internalized AMPA receptors were detected as described by Wei et al.^23^ Briefly, surface GluR1 was labeled with a polyclonal anti‐GluR1 antibody (1:100; Millipore, 07‐660) in living cells for 20 minutes at 37°C in the culture medium followed by exposure to OGD or normoxia. After washing, the antibody that binds to the remaining surface GluR1 was stripped off with an acid solution (0.5 mol/L NaCl, 0.2 N acetic acid) at 4°C for 4 minutes. Cells were then washed, fixed, permeabilized, and incubated with a monoclonal anti‐GluR1 antibody (1:200; Santa Cruz Biotechnology, sc‐13152) for 2 hours at room temperature. The internalized GluR1 (labeled with a polyclonal GluR1 antibody) was detected with the Alexa Fluor 594 (red)–conjugated antibody, whereas the total GluR1 (labeled with a monoclonal GluR1 antibody) was detected with Alexa Fluor 488 (green)–conjugated secondary antibodies.

Double‐live immunostaining of primary hippocampal cultures with NP1 was done as described by O'Brien et al.^2,7^ Briefly, hippocampal neurons were live‐labeled with both anti‐NP1 (1:100; BD Transduction Laboratories, Temecula, CA) and anti‐GluR1 (1:100; Millipore) for 45 minutes at 37°C. Neurons were then fixed with 3.7% formaldehyde, and permeabilized cells were stained with appropriate Alexa Fluor–conjugated secondary antibodies (Invitrogen). For the detection of AMPA receptors at synapses, neurons were fixed, permeabilized, and stained with a polyclonal anti‐GluR1 antibody (1:500) and a polyoclonal anti‐PSD‐95 antibody (1:300; Santa Cruz Biotechnology, sc‐6926).

For triple‐label experiments, neurons were fixed, but not permeabilized, and stained with anti‐NP1 (1:200). After washing, neurons were permeabilized and incubated with a polyclonal anti‐GluR1 antibody (1:500) and a polyclonal anti‐PSD‐95 antibody (1:300; Santa Cruz Biotechnology).

### SDS/PAGE, Western Blot, and Biochemical Measurement of Surface‐Expressed Receptors

Western blotting with whole‐cell lysates was performed with standard methods as described previously.^16,21–22^ Blots were probed with primary antibodies for phospho‐GluR1 (Ser‐845; 1:1000; Novus Biologicals, Littleton, CO), GluR1 (1:1000; Millipore), and β‐actin (1:5000; Sigma). Horseradish peroxidase (HRP)–conjugated secondary antibodies (GE Healthcare) were used at 1:5000 dilutions for 1 hour at room temperature. HRP reaction product was then visualized by enhanced chemiluminescence using an ECL Western blotting detection kit (Pierce, Rockford, IL). Digitized images were quantified using NIH ImageJ software.

The surface protein cross‐linking assay was used as described by Archibald et al.^24^ Briefly, after vehicle and OGD treatment, the cultures were washed twice with warm PBS and incubated with 1 mg/mL BS^3^ (bis[sulfosuccinimidyl] suberate) for 10 minutes at 4°C (Pierce Biotechnology). The cells were washed with ice‐cold quenching buffer and harvested. As a negative control, another set of cultures was collected in the same way without BS^3^ exposure. In the absence of BS^3^, anti‐GluR1 antibodies detect only 1 immunoreactive band; in the presence of BS^3^, they detect 2 bands, the upper‐surface GluR1, and the lower intracellular GluR1 signals. The BS^3^ treatment did not affect β‐actin signals.

### Coimmunoprecipitation and Immunoblotting

Coimmunoprecipitation experiments were performed as described previously.^1,16^ Briefly, 100 μg of total proteins was subjected to immunoprecipitation by incubation overnight with 1 μg of GluR1 polyclonal antibody (Millipore) at 4°C with constant shaking. Then, 20 μL of protein A/G‐agarose conjugated beads (Santa Cruz Biotechnology) was added to each sample and incubated for 2 to 4 hours at 4°C. The beads were collected, washed, and boiled for 3 minutes in 50 μL of 1× electrophoresis sample buffer, followed by Western blot analysis.

### Microscopy and Image Analysis

Identification of GluR1 clusters and their colocalization with NP1 and PSD‐95 was performed using an inverted fluorescence microscope (Olympus IX51 fitted with DP2‐DSW‐V3.2 application software) at ×10 magnification and ZEISS Axioimager M2 (AxioVision SE64 Rel. 4.8.1 application software) at ×63 magnification. Images were obtained with the same background and parameters. Images were manually thresholded, and because of intense nonspecific binding on the cell body, clusters in well‐defined proximal dendrites 5 μm from the soma were counted, and averages were calculated per 20 μm.^23^ Colocalization was calculated using an Image J colocalization plugin (http://rsb.info.nih.gov/ij/plugins/colocalization.html). Regarding quantitative analysis, raw images (fixed exposures and no postprocessing) were normalized to 8‐bit images. All values are shown as mean±SEM, n=23 to 25. The sample size (n) indicates the segment number of proximal dendritic processes (does not include soma, 2 to 3 segments/neuron). Three to 4 independent experiments for each of the groups were performed.

### Statistical Analysis

Statistics were performed using GraphPad Prism version 5.0 program (GraphPad Software). For image analyses between 1 experimental group and 1 control group, the 2‐tailed Student *t* test was used to determine if differences existed between means. All cytotoxicity, cell viability, and Western blot data involving multiple groups were analyzed by the nonparametric Kruskal–Wallis test. Data are presented as mean±SEM and regarded as statistically significant at *P<*0.05.

## Results

### NP1 Induction and Neuronal Death in Primary Hippocampal Neuronal Cultures Following Exposure to Oxygen Glucose Deprivation

**Figure 1.**
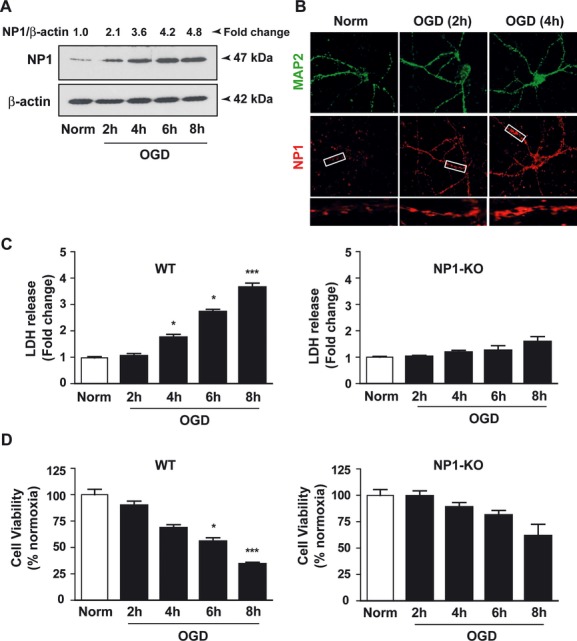
NP1 induction is associated with hippocampal neuronal death exposed to OGD. A, Total cellular proteins were analyzed by SDS‐PAGE and immunoblotted for NP1 protein. Quantitative densitometry values normalized to β‐actin (NP1/β‐actin) are also shown. B, Immunostaining of cultured hippocampal neurons with NP1‐specific antibody showed significantly enhanced immunoreactivity following OGD. Primary hippocampal neuronal death and viability were measured by LDH release (C) and 3‐(4,5‐dimethylthiazol‐2‐yl)‐2,5‐diphenyl tetrazolium bromide (MTT) reduction (D) assays, respectively, in control normoxia and OGD (2 to 8 hours) conditions. Quantification of LDH release and MTT reduction showed significantly increased cell death in WT neurons compared with normoxia controls. In contrast, *NP1* gene deletion prevented OGD‐induced hippocampal neuronal death compared with that of WT cultures. Data represent mean±SEM (n=6, the number of independent observations in an experiment; *P<0.05; ***P<0.001). Each experiment was performed 3 to 4 times. NP1 indicates neuronal pentraxin 1; OGD, oxygen glucose deprivation; LDH, lactate dehydrogenase; WT, wild‐type.

Primary neuronal cultures provide an excellent model for investigating cellular and molecular signaling cascades that regulate cell fate—death and survival.^25^ First, we examined NP1 induction and neuronal death in mature hippocampal neuronal cultures (DIV 12 to 14) following exposure to oxygen glucose deprivation (OGD) for 2 to 8 hours. Increased induction of NP1 protein levels was observed in hippocampal neurons after exposure to OGD (Figure 1). Western blot analysis of total cellular extracts showed an OGD time‐dependent increase in NP1 protein levels with an ≈2‐fold induction observed at 2 hours of exposure, which reached the maximum at 8 hours compared with normoxic control cultures (Figure 1A). Immunocytochemical experiments also showed enhanced NP1‐specific immunofluorescence in the hippocampal neurons at the dendritic sites after OGD exposure (2 to 4 hours) (Figure 1B), confirming NP1 induction in response to OGD.

Next, to correlate between NP1 induction and cell death, we measured cell cytotoxicity by LDH release and cell viability by MTT reduction assays in WT and NP1−/− hippocampal cultures exposed to OGD (2 to 8 hours). OGD caused a time‐dependent increase in cell death, as evidenced by both LDH release and the MTT reduction assay (Figure 1). Quantitative estimation revealed that OGD caused ∼50% to 60% neuronal death (at 6 to 8 hours) in WT hippocampal neurons compared with the normoxia controls (Figure 1C and 1D), which is consistent with our previous report.^21–22^ To demonstrate whether NP1 induction is directly contributing to the observed neuronal death, we used NP1−/− neurons exposed to identical OGD conditions. Our results showed very negligible changes (not significant) in LDH release and MTT reduction in NP1−/− cultures compared with that observed in WT neurons and normoxia controls (Figure 1C and 1D). Our results suggest that NP1−/− neurons are significantly protected against OGD.

### OGD Causes Increase in Surface Expression With Simultaneous Decrease in Internalization of AMPAR Subunit GluR1 and Enhanced GluR1 Synaptic Clustering

**Figure 2.**
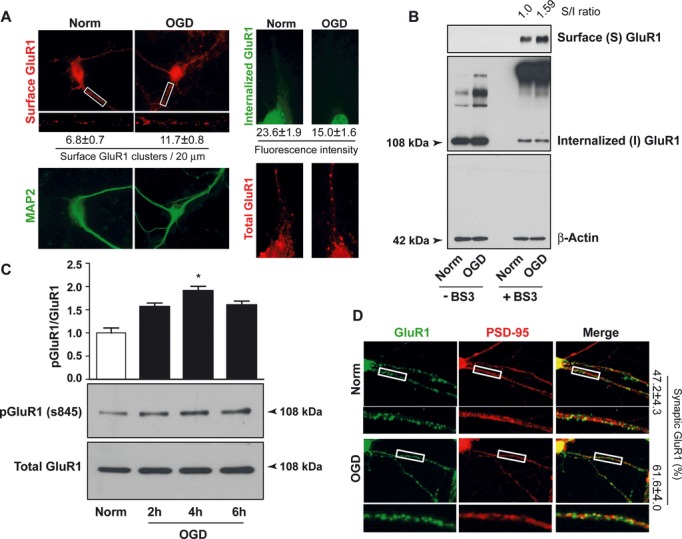
OGD enhances GluR1 surface expression with simultaneous reduction in receptor internalization and increases GluR1 synaptic localization. A, Immunocytochemical images of surface (left) and internalized (right) GluR1 in hippocampal neurons following OGD (2 hours). Quantitative analyses of surface GluR1 (*P*<0.01) and fluorescence intensity of internalized GluR1 (*P*<0.05) are shown at the bottom of the corresponding images. B, Experiments were performed in the presence and absence of Bis[sulfosuccinimidyl] suberate (BS^3^) after OGD (4 hours). The blots were probed for GluR1 followed by reprobing with β‐actin antibody. C, Western blot showing phospho‐GluR1 (Ser‐845) and total GluR1 bands with their respective quantitative densitometry (mean±SEM, n=3, the number of repeat experiments; *P<0.05). D, Immunocytochemical images of synaptic GluR1 clusters (PSD‐95 colocalized, yellow puncta) in hippocampal cultures exposed to either normoxia or OGD (2 hours). Quantitative analyses showing the percentage of the GluR1/PSD‐95 colocalization are depicted in the respective images (*P*<0.05). OGD indicates oxygen glucose deprivation; MAP2, microtubule‐associated protein 2; PSD, postsynaptic density.

Neuronal injury from cerebral hypoxia‐ischemia has been attributed to overstimulation of the NMDA and AMPA subtypes of glutamate receptors.^17–18,25^ To test whether the changes in the number of available AMPARs on the cell surface can be accounted for by OGD‐induced neuronal death, we carried out quantitative surface immunostaining of GluR1 (Figure 2). Exposure to OGD caused a significant increase in surface GluR1 cluster density (number of clusters/20 μm of dendrites; normoxia, 6.8±0.7; n=24; OGD, 11.7±0.8; n=25; *P<*0.01; Figure 2A). Next, we detected AMPAR internalization as described in the Methods section. We observed a significant decrease in the fluorescence intensity of the internalized GluR1 on neuronal dendrites (normoxia, 23.6±1.9; n=23; OGD, 15.0±1.6;, n=23; *P<*0.05) following OGD.

To further demonstrate the change in surface AMPARs, we performed surface protein cross‐linking experiments with BS^3^ to measure levels of surface GluR1. OGD exposure of hippocampal neurons significantly increased surface GluR1, whereas the internal control actin band remaining unchanged (Figure 2B). Next, we asked how surface GluR1 levels increase in response to OGD. We found increased phosphorylation of GluR1 (Ser‐845) following OGD (2 to 6 hours), whereas total GluR1 level remained unchanged (Figure 2C). To provide more direct evidence on AMPARs trafficking at synapses, we measured GluR1 clusters colocalized with the synaptic marker PSD‐95. The percentage of the GluR1/PSD‐95 colocalized structures of total PSD‐95 was calculated. A significant increase in synaptic GluR1 cluster density was observed following OGD (normoxia, 47.2±4.3%, n=25; OGD, 61.6±4.0%, n=24; *P<*0.05), whereas total GluR1 or PSD‐95 clusters were not altered by OGD exposure (Figure 2D), indicating an increase in synaptic GluR1.

### OGD Enhances Interaction Between NP1 and GluR1 at the Synapses

**Figure 3.**
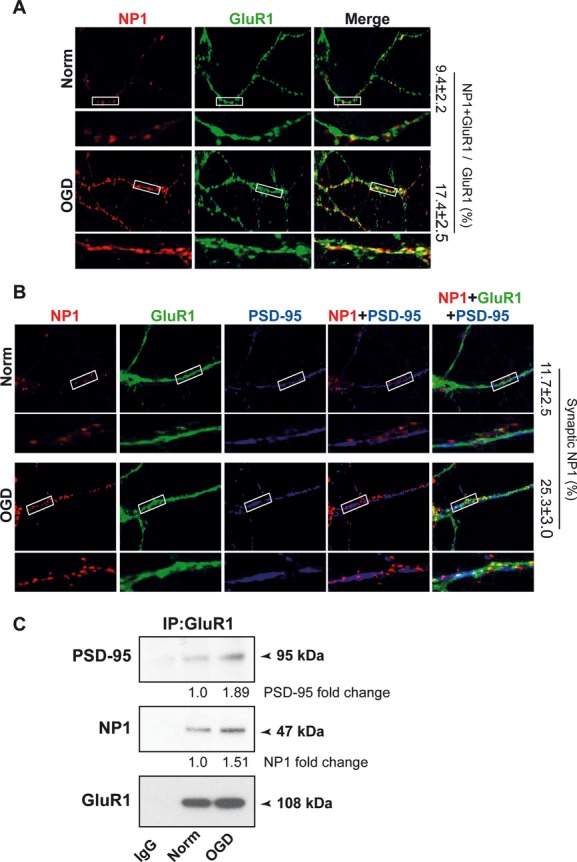
OGD enhances interaction between NP1 and GluR1 at synaptic sites. A, Live immunostaining of hippocampal neurons with NP1 and GluR1 following OGD (2 hours). Quantitative analyses show the percentage of the NP1‐GluR1 colocalized clusters in the respective images (*P*<0.05). B, NP1 (red), GluR1 (green), and PSD‐95 (blue) colocalize at hippocampal dendrites. Quantitative analyses show the percentage of the colocalization of NP1 and PSD‐95 (synaptic NP1) (*P*<0.01). C, WB of GluR1 immunoprecipitates show increases in NP1 and PSD‐95 co‐precipitation in response to OGD. Quantification of the band intensity is depicted at the bottom of the blots. OGD indicates oxygen glucose deprivation; NP1, neuronal pentraxin 1; WB, Western blot; PSD, postsynaptic density.

Next, we examined the relationship between NP1 induction, and surface expression and synaptic clustering of GluR1 in response to OGD (Figure 3). Live immunostaining with both NP1 and GluR1 antibodies revealed a significant increase in NP1‐GluR1 colocalized clusters in OGD‐exposed neurons compared with normoxic controls (Figure 3A). The percentage of NP1/GluR1 coclusters versus PSD‐95 puncta was found to be significantly higher following OGD (normoxia, 9.4±2.2%, n=24; OGD, 17.4±2.5%, n=23; *P<*0.05). To further investigate the interaction between NP1 and GluR1 at synapses, we performed triple labeling with antibodies specific to NP1, GluR1, and PSD‐95. Fluorescence microscopy showed a much higher degree of colocalization of NP1‐GluR1 at the synaptic sites after OGD, as evidenced by the higher percentage of NP1/PSD‐95 colocalization (normoxia, 11.7±2.5%, n=25; OGD, 25.3±3.0%, n=25; *P<*0.01; Figure 3B). To support our immunofluorescence findings, we measured GluR1‐bound NP1 and PSD‐95 by coimmunoprecipitation experiments. Total cellular extracts of primary hippocampal neurons exposed to either OGD or normoxia were immunoprecipitated with GluR1 antibody. NP1 coprecipitated with GluR1, and OGD exposure significantly increased GluR1‐bound NP1 protein levels (Figure 3C). Similar to NP1, we found that GluR1 bound to the PSD‐95 and that GluR1‐bound PSD‐95 levels increased significantly in response to OGD (Figure 3C). Together, our findings clearly indicate enhanced synaptic interaction between NP1 and GluR1 following OGD.

### NP1 Is Required for OGD‐Induced Surface GluR1 Expression and Synaptic Localization

**Figure 4.**
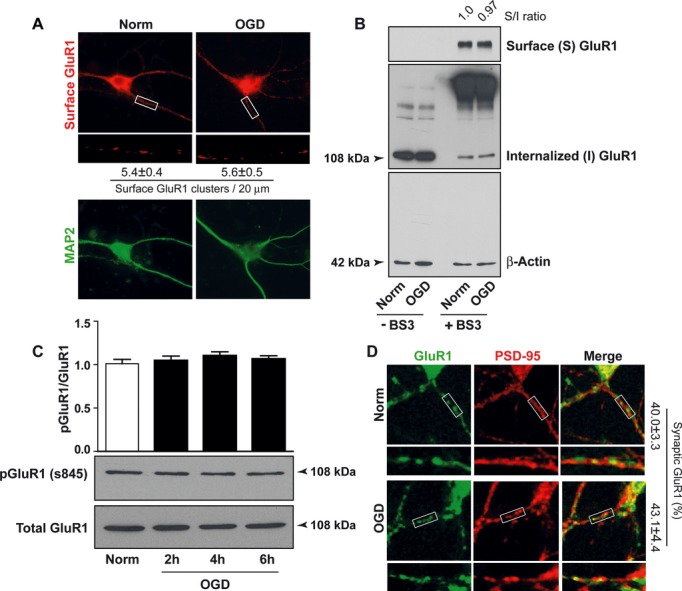
NP1 deletion prevents OGD‐induced increase of surface GluR1 clusters and their synaptic localization. A, Immunocytochemical images of surface GluR1 staining in NP1‐KO hippocampal neurons following OGD (2 hours). Quantification of surface GluR1 is shown at the bottom of the respective images. B, Experiments were performed in the presence and absence of BS3. The extracted proteins were scored for GluR1 and β‐actin, respectively. C, Total cellular proteins were immunoblotted for phosphorylated GluR1 (Ser‐845) and total GluR1 signals. Quantitative densitometry (mean±SEM, n=3, the number of repeat experiments) is also shown. D, Immunocytochemical images of synaptic GluR1 clusters (PSD‐95 colocalized, yellow puncta) in NP1‐KO hippocampal cultures. Quantitative analyses show the percentage of the colocalization of GluR1 clusters and PSD‐95 in the respective images. NP1 indicates neuronal pentraxin 1; OGD, oxygen glucose deprivation; KO, knockout; PSD, postsynaptic density.

To directly demonstrate whether NP1 mediates OGD‐induced enhanced surface GluR1 expression and synaptic clustering, we used NP1−/− hippocampal neurons for quantitative surface immunostaining after OGD (Figure 4). Strikingly, NP1 deletion blocked the increase of surface GluR1 cluster density following OGD (number of clusters/20 μm of dendrite; normoxia, 5.4±0.4, n=25; OGD, 5.6±0.5, n=24; Figure 4A). Surface protein cross‐linking experiments using BS^3^ also showed no increase in surface GluR1 levels in NP1−/− neurons (Figure 4B), clearly indicating a critical role for NP1 in GluR1 surface expression. To further support our findings, we observed no significant differences in both phospho‐GluR1 and total GluR1 levels between normoxia controls and OGD‐exposed NP1−/− neurons (Figure 4C). Next, to provide more direct evidence of NP1 involvement in synaptic AMPAR trafficking, we measured the synaptic GluR1 clusters in NP1−/− neurons. We observed no significant change in synaptic GluR1 (colocalized with PSD‐95) cluster density in OGD‐exposed cultures (normoxia, 40.0±3.3%, n=23 versus OGD, 43.1±4.4%, n=24; Figure 4D). Our results suggest that NP1 is required for OGD‐induced increase in synaptic clustering of GluR1.

### NP1 Gene Deletion Protects Against OGD‐ and AMPA‐Induced Neuronal Death

**Figure 5.**
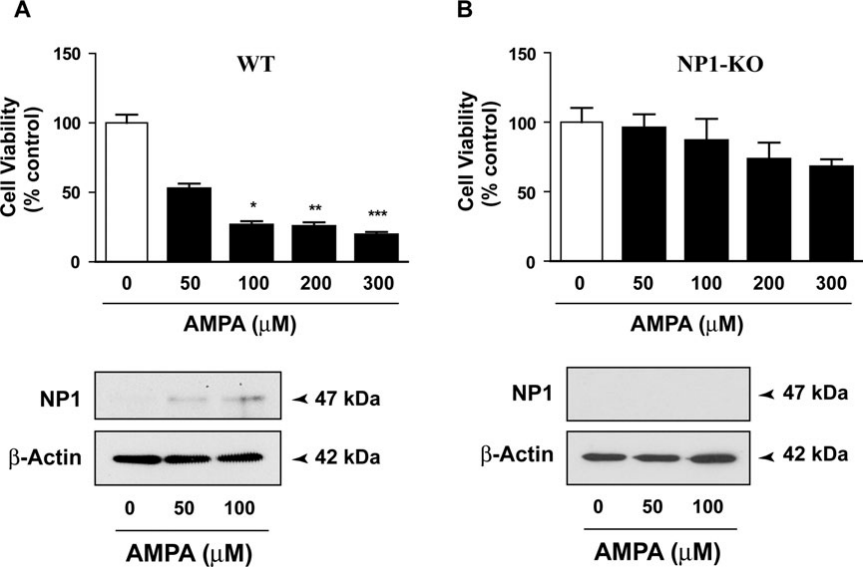
NP1 is associated with AMPA‐induced excitotoxicity in hippocampal cultures. Primary hippocampal neuronal cultures from WT (A) and NP1‐KO (B) were exposed to various concentrations of AMPA (50 to 100 μmol/L) for 24 hours. AMPA treatment induced cell death and NP1 induction in WT cultures but not in NP1‐KO cultures. Data are presented as mean±SEM (n=6, the number of independent observations in an experiment; *P<0.05; **P<0.01; ***P<0.001; each experiment performed 3 to 4 times). NP1 indicates neuronal pentraxin 1; AMPA, α‐amino‐3‐hydroxy‐5‐methyl‐4‐isoxazole propionic acid; WT, wild type; KO, knockout.

To directly test whether NP1 affects AMPA‐mediated neuronal death, hippocampal neurons were exposed to different concentrations of AMPA (50 to 300 μmol/L) for 24 hours as described previously (Figure 5).^16^ We found 100 μmol/L of AMPA caused >60% decrease in cell viability (Figure 5A, top panel) and a robust increase in NP1 expression (Figure 5A, bottom panel) in WT neurons compared with controls. In contrast, the MTT viability assay revealed significant neuroprotection against AMPA‐induced neuronal death in NP1−/− neurons, similar to that observed after OGD (Figure 5B, top panel), although at higher concentration of AMPA (300 μmol/L), cell viability was slightly decreased. In addition, Western immunoblotting using NP1‐specific antibody validated the genetic deletion of NP1 in knockout cultures used in this study. There was no induction of the NP1 protein following AMPA treatment (Figure 5B, bottom panel). Our results suggest that NP1 is directly involved in AMPAR‐mediated hippocampal neuronal death.

**Figure 6.**
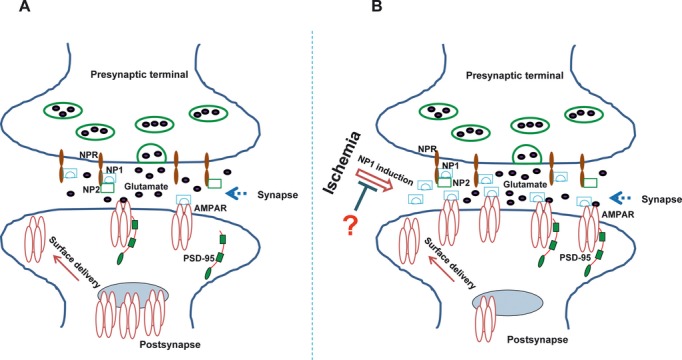
NP1 modulation of synaptic AMPAR trafficking in ischemia: a working hypothesis. A, Under normoxic condition, glutamate mediates essential excitatory synaptic transmission by binding to cognate AMPARs. Low levels of NP1 and NP2, in association with NPR, can also form complexes with AMPA GluR1 to facilitate synaptic transmission. B, Robust increase in NP1 following ischemia translates into more binding to GluR1 and enhanced interaction at the synaptic sites. The uninhibited NP1–GluR1 interaction could further enhance surface delivery of the receptors, resulting into overactivation of the receptors and excitotoxicity. Inhibition of NP1 induction could limit OGD‐ and AMPAR‐mediated excitotoxicity. NP1 indicates neuronal pentraxin 1; AMPAR, α‐amino‐3‐hydroxy‐5‐methyl‐4‐isoxazole propionic acid receptor; NP2, neuronal pentraxin 2; NPR, neuronal pentraxin receptor; OGD, oxygen glucose deprivation.

To further demonstrate the specific involvement of NP1 in AMPAR‐mediated downstream functions, we examined mitochondrial translocation of a prodeath protein, Bad, involved in glutamate‐induced excitotoxic neuronal death^26–27^ and reported to be a downstream signaling event elicited by AMPAR.^28^ We performed subcellular fractionations in normoxia control and OGD‐exposed WT and NP1−/− hippocampal neurons. In cytosolic fractions of WT neurons, significantly decreased levels of Bad were observed with a concurrent increase in the mitochondrial fraction following OGD compared with normoxia controls. In contrast, no significant change in Bad protein levels was observed in both the cytoplasmic and mitochondrial fractions from NP1−/− hippocampal neurons compared with normoxia control cultures (Figure S1). Our results suggest that NP1 directly modulates AMPAR‐elicited downstream excitotoxic signaling events (Figure 6).

## Discussion

The role of AMPA receptor trafficking in synaptic plasticity under physiological conditions is well established; however, its role in ischemia‐induced synaptic remodeling and/or neuronal death is unclear. In the present study, we show direct NP1‐regulation of surface GluR1 expression and synaptic clustering of NP1 with GluR1 in hippocampal neurons. OGD exposure promotes redistribution of GluR1 at the postsynaptic membrane, significantly increasing NP1–GluR1 interaction and ischemic neuronal death. We also found that this clustering activity involves physical association between NP1 and GluR1 and that NP1 exhibited profound synaptic coclustering with GluR1 following OGD. The most important findings in this report are the knocking down of NP1 gene (*Ntpx1*) blocked OGD‐induced enhanced surface GluR1 expression, its synaptic clustering, and both OGD‐ and AMPA‐mediated neuronal death. These results indicate that NP1 plays a critical role in surface clustering of AMPAR GluR1 at excitatory synapses and excitotoxicity‐mediated ischemic neuronal death.

Hypoxic‐ischemic neuronal injury is triggered by the activation of the glutamatergic excitotoxic cascade^6^ and several downstream cytotoxic pathways.^4,17^ At excitatory synapses of central neurons, PSD‐95 couples AMPA receptors to intracellular proteins necessary for synaptic targeting of AMPARs.^11^ Also, synaptic activation requires surface delivery of AMPARs.^29^ We found enhanced surface clustering of GluR1 with a simultaneous decrease of internalized GluR1 on the dendrites of hippocampal neurons after OGD and significantly increased neuronal death. It is known that GluR1 surface insertion is regulated by phosphorylation of GluR1 at Ser‐845.^30^ Thus, the increased levels of phospho‐GluR1 (Ser‐845) observed in this study further corroborate enhanced surface GluR1 trafficking in response to OGD. This surface localization of GluR1 was also documented by surface cross‐linking with BS^3^, showing higher levels of surface GluR1. NP1, which is closely related to Narp, has been reported to play a role in excitatory synaptic plasticity in developing and adult brain, and both NP1 and NP2 selectively accumulate at excitatory synapses in primary hippocampal cultures.^31^ We found that NP1 colocalizes and physically associates with GluR1 and that OGD significantly enhances NP1–GluR1 interactions at synaptic sites, as evident by increased NP1‐GluR1‐PSD‐95 colocalization and coimmunoprecipitation of NP1 with GluR1 and PSD‐95. It appears that OGD recruits NP1 protein to GluR1 subunits to form clusters at excitatory synapses and that increased NP1–GluR1 interaction sensitizes neurons to OGD‐ and AMPA‐induced neuronal death. Our results clearly indicate specific involvement of NP1 in controlling synaptic clustering of GluR1 and excitotoxic function.

The family of long pentraxin proteins has several characteristics: ability to form side‐to‐side and head‐to‐head multimeric aggregates and to bind other proteins via a lectin‐like domain.^32^ Unlike PSD‐95, NP1 is an extracellular protein with no PDZ domains and no access to the intracellular domains on AMPAR subunits.^7,32^ This suggests that NP1 interacts with extracellular domains on the receptor proteins. Sia et al showed that knockdown of both NP1 and NPR decreases axonal ability to recruit AMPA GluR4,^33^ and knockout of NP1 significantly reduced NPR levels, suggesting dependence on each other for synaptic stability.^15^ We propose that presynaptic NPR binds to NP1, allowing NP1 to trans‐synaptically attach to the extracellular domain of GluR1 at the postsynaptic specialization and thereby facilitating glutamate binding and enhancing excitotoxicity. In contrast, NP1−/− neurons showed reduced excitotoxicity by limiting synaptic GluR1 cluster formation. Xu et al suggested that NP1 and NP2 cofunction to induce AMPAR aggregation and integration into NP1/NP2 heteromultimers, which have a greater ability to induce receptor coclustering.^31^ Furthermore, NP1 has also been reported to participate in AMPAR GluR4 recruitment in developing postsynaptic specialization.^33^ Thus, it appears that formation of NP2 clusters alone and NP1–NP2 heterocomplexes with GluR1 may be required for physiological functions in developmental and activity‐dependent synaptic plasticity, but not for pathological brain injury mechanisms. Therefore, differential regulation of NP1 and NP2 provides a mechanism by which neuronal cells can tune their expression and the magnitude of AMPAR clustering during physiological functions and in pathological conditions.

Although excessive activation of glutamate receptors mediates ischemic brain injury, glutamate receptors also mediate essential neuronal excitation under physiological conditions to maintain normal neuronal functions.^4,17–18^ Most glutamate receptor antagonists indiscriminately block essential glutamate functions and have failed in clinical trials despite their therapeutic potential.^34^ Most recently, Aarts et al suggested a potential treatment option for ischemic brain damage by perturbing NMDA receptor–PSD‐95 interactions rather than directly blocking NMDARs.^20^ However, PSD‐95 also plays an equally important role in synaptic plasticity and development in the brain by regulating AMPAR trafficking under normal physiological conditions.^17–18,20^ Thus, complete blockade of interactions between NMDAR and PSD‐95 is therapeutically impractical. In this study, we show that NP1 interacts with both GluR1 and PSD‐95 and enhances synaptic clustering of GluR1, leading to excitotoxicity and ischemic neuronal death, whereas NP1‐KO neurons were protected against AMPA‐induced neuronal death, demonstrating a direct link between NP1 function and AMPAR‐mediated cell death. Thus, interfering with NP1 and GluR1 interactions would neither compromise AMPAR‐mediated essential neuronal functions nor completely abolish AMPAR–PSD‐95 interactions; rather, it would interrupt signaling downstream of AMPAR, which leads to neuronal death. It is probable that suppression of NP1 induction by suitable pharmacological compounds might hold potential for treating stroke and other diseases, which is a subject of our ongoing investigations.

In summary, our study points to a novel mechanism by which NP1 regulates synaptic clustering of GluR1, an event that has profound effects on signaling pathways downstream of AMPAR activation and excitotoxicity and on ischemic neuronal death. Genetic deletion of *NP1* restricts recruitment of GluR1 at synapses, thereby preventing AMPA‐mediated excitotoxicity and ischemic neuronal death. Our results reveal a critical role of NP1 in modulating synaptic AMPAR clustering and its efficacy after OGD, suggesting NP1 as a practical target for preventing ischemic neuronal injury.
